# Effects of long-term low-level radiation exposure after the Chernobyl catastrophe on immunoglobulins in children residing in contaminated areas: prospective and cross-sectional studies

**DOI:** 10.1186/1476-069X-13-36

**Published:** 2014-05-10

**Authors:** Daria M McMahon, Vitaliy Y Vdovenko, Wilfried Karmaus, Valentina Kondrashova, Erik Svendsen, Oksana M Litvinetz, Yevgenia I Stepanova

**Affiliations:** 1 Arnold School of Public Health, University of South Carolina, 800 Sumter Street, Columbia, SC 29208, USA; 2 Research Center for Radiation Medicine, Academy of Medical Sciences of Ukraine, 53 Melnikova St, Kiev 04050, Ukraine; 3 Division of Epidemiology, Biostatistics, and Environmental Health Science, School of Public Health University of Memphis, 301 Robison Hall, Memphis 38152 TN, USA; 4 Tulane University School of Public Health and Tropical Medicine, 1440 Canal Street, New Orleans 70112 LA, USA

**Keywords:** ^137^Caesium, Chernobyl, Epidemiology, Immunoglobulin, Ionizing radiation, Children

## Abstract

**Background:**

After the Chernobyl nuclear incident in 1986, children in the Narodichesky region, located 80 km west of the Chernobyl Power Plant, were exposed to ^137^Cesium (^137^Cs). Little is known about the effects of chronic low-level radiation on humoral immune responses in children residing in contaminated areas.

**Methods:**

In four different approaches we investigated the effect of residential ^137^Cs exposure on immunoglobulins A, G, M, and specific immunoglobulin E in children. In a dynamic cohort (1993–1998) we included 617 children providing 2,407 repeated measurements; 421 and 523 children in two cross-sectional samples (1997–1998 and 2008–2010, respectively); and 25 participants in a small longitudinal cohort (1997–2010). All medical exams, blood collections, and analyses were conducted by the same team. We used mixed linear models to analyze repeated measurements in cohorts and general linear regression models for cross-sectional studies.

**Results:**

Residential soil contamination in 2008 was highly correlated with the individual body burden of ^137^Cs. Serum IgG and IgM concentrations increased between 1993 and 1998. Children with higher ^137^Cs soil exposure had lower serum IgG levels, which, however, increased in the small cohort assessed between 1997 and 2010. Children within the fourth quintile of ^137^Cs soil exposure (266–310 kBq/m^2^) had higher IgM serum concentrations between 1993 and 1998 but these declined between 1997 and 2010. IgA remained stable with median ^137^Cs exposures related to higher IgA levels, which was corroborated in the cross-sectional study of 2008–2010. Specific IgE against indoor allergens was detected less often in children with higher ^137^Cs exposure.

**Conclusions:**

Our findings show radiation-related alterations of immunoglobulins which by themselves do not constitute adverse health effects. Further investigations are necessary to understand how these changes affect health status.

## Background

As a result of the Chernobyl nuclear incident on April 26, 1986, the Narodichesky district (Jitomirsky region, Ukraine), located about 80 km west of Chernobyl Nuclear Power Plant, was contaminated with different radionuclides. A predominant radionuclide was ^137^Cs with a half-life of 30 years. According to statistics from the only All-Ukrainian population census, 24 years after the Chernobyl catastrophe, the population of Narodichi consisted of approximately 11,400 people, including 2,000 children [1]. The Narodichesky district is a farming area; its ^137^Cs soil contamination in 1992 varied between 59 and 879 kBq/m^2^ and in 2011 between 16 and 488 kBq/m^2^[2]. The region covers 1284 square kilometers (diameter 60–80 km) and has the same whether, wind and climate conditions.

One of the important questions is whether radiation following the disaster affected immune responses. A report of the UN Chernobyl Forum stated that “reported immunological effects of radiation exposure from the Chernobyl incident appeared to be related to both changes in the amount or function of peripheral lymphocytes and changes in serum immunoglobulin levels” [3]. Few studies have been conducted on the link between radiation and immune markers [4-13]. A recently published report [14] concluded that the immune status is imbalanced in children whose parents were evacuated as children from either the city Pripyat adjacent to the reactor or from the 30-km zone surrounding the Chernobyl Nuclear Power Plant (first zone) [15]. Also, the immune system in children whose parents resided in the third and second zones was characterized by similar changes [14]. A recent review [16] stated the need for further investigations into the long term effects of low dose ^137^Cs irradiation on the immune system. Our work herein focuses on immunoglobulins (Ig) A, G, M, and E in children from the Narodichesky district.

After the Chernobyl incident, serum levels of IgA in children aged 1–14 years varied with time and exposure. Within the first 1.5 months after the incident, in children 7–14 years residing in villages of Gomel and Mogilev, Belarus, with density of ^137^Cs soil contamination over 1 Ci/km^2^ (Note: 1 curie (Ci)/km^2^ = 37 kBq/m^2^), serum IgA was significantly higher compared to non-exposed controls [6,9,11] and returned back to normal levels within seven months [9]. During the first six years after the Chernobyl incident serum IgA concentrations in children were slightly higher than in controls but did not exceed the normal limits [6]. From 1986 to 1992 serum IgA levels decreased significantly in two groups with low to medium levels of soil contamination, 3.46 Ci/km^2^ (~128 kBq/m^2^) and 6.04 Ci/km^2^(~223 kBq/m^2^) [6]. Similar changes were detected in children from Brest, Gomel, and Mogilev (Belarus) residing in areas with high levels of radiation (80–120 Ci/km^2^) nine years after the incident [5]. Another study linked serum immunoglobulins to internal doses of ^137^Cs in children, accumulated over 14 years after the Chernobyl incident [12]. In the study by Chebanenko et al. [12] whole body concentration (WBC) of ^137^Cs was measured in Bq using gamma-spectrometer (Whole Body Counter SKRINNER-3M). This measurement was used for calculation of the internal dose of ^137^Cs in mSv. According with their WBC, children were divided into 3 groups: I - 1,087-13,956 Bq (mean 4,540 ± 210 Bq), internal dose 0.150-1.147 mSv (mean 0.412, mSv); II - 1,046-10,060 Bq (mean 3,820 ± 260 Bq), internal dose 0.106-0.899 mSv (mean 0.209 mSv); III - 0–960 Bq (mean 410 ± 60 Bq), internal dose was not reported. Decreased levels of IgA occurred more frequently at higher internal doses. In summary, there is a lack of studies and inconsistent findings on whether long-term radiation exposure affects serum IgA concentrations.

Serum concentrations of IgG in children ages 7–14 years residing in villages of Gomel and Mogilev with soil density of ^137^Cs exceeding 1 Ci/km^2^ were significantly decreased within the first 45 [9] and 90 days [6] after the Chernobyl incident. IgG concentrations returned to the level of unexposed controls within the following seven months [9]. Serum levels of IgG in children ages 3–7 years residing in contaminated areas in Gomel and Mogilev, Belarus, started to increase a year after the incident [17]. In agreement with this “suppressed-increased time pattern”, Titov et al (1995) reported for the same areas of Belarus that during the first six years after the incident, IgG serum concentrations in children 1–14 years of age increased over time, reached levels of the controls in 1990, and then exceeded them in 1992. From 1986 to 1992, serum levels of IgG were reported to be positively correlated with the levels of ^137^Cs soil contamination [6]. Analysis of IgG subclasses during the phase of increase (1993) demonstrated that concentrations of IgG1 and IgG4 were positively correlated with ^137^Cs body burden whereas for the concentrations of IgG2 and IgG3 subclasses correlations were inversely associated [6]. Fourteen years after the incident, the proportion of children with hypoimmunoglobulinemia G was 12.4% in the group with the lowest body burden (0–960 Bq), 14.3% in children with a body burden between 1,046 Bq and 10,060 Bq, and 11.1% in the highest group (1,087-13,956 Bq) [12]. In summary, prior findings suggest that IgG seems to follow a “suppressed-increased time pattern” after the exposure to ionizing radiation.

During the first 1.5 months after the Chernobyl accident IgM serum levels in children 1–14 years residing in the Braginsky region of Belarus with a ^137^Cs soil contamination density of 3 Ci/km^2^ (111 kBq/m^2^) seemed to be slightly increased [7,9]. However, another study with a larger sample (n = 6,000), showed that immediately after the fallout serum concentrations of IgM in children ages 7–14 years residing in villages of Gomel and Mogilev, Belarus, with density of soil contamination with ^137^Cs over 1 Ci/km^2^ started to decrease and were the lowest by day 30 and then recovered [6]. By day 90 after the Chernobyl incident IgM levels exceeded the concentrations found in controls. In the same study, levels of serum IgM in children 1–14 years kept increasing during the first six years after the Chernobyl. Hence, in 1992 the levels were significantly higher than in controls in all age groups [6]. Ten years after the incident, children 1–14 years with frequent respiratory infections residing in contaminated territories had elevated levels of IgM in serum and saliva [8]. In children residing in Gomel and Mogilev, Belarus, levels of IgM were positively correlated with levels of ^137^Cs soil contamination [6]. In another study conducted in Brest, Gomel and Mogilev, children residing in areas with radiation levels of 37–185 kBq/m^2^ had increased serum IgM; however, at high levels (2,960-4,400 kBq/m^2^) concentrations of IgM were decreased [5,17]. Fourteen years after the Chernobyl incident, the proportion of children with IgM levels below clinical references positively correlated with internal ^137^Cs dose [12]. In the same study, increased IgM levels were consistently observed in 10% of children and did not vary by the internal ^137^Cs dose. In summary, no clear response pattern of IgM after exposure to radiation was seen in prior studies.

IgE exists mostly in a bound state with mast cells located beneath the skin and mucosa. The binding of IgE with antigens causes degranulation of mast cells, which then can induce an allergic reaction. It was reported that between 1987–1995, approximately 40% of children residing in contaminated territories had increased levels of total IgE in serum [9,18]. In 1993, concentrations of total IgE were 2–3 times higher than clinical standards [7]. Between 1996 and 1999, in 12 children residing in contaminated areas with ^137^Cs in the soil 260–370 kBq/m^2^ serum IgE levels were significantly increased (mean > 485 IU/ml) compared to 108 control children from clean areas, with ^137^Cs soil levels of 0.037 kBq/m^2^ (mean: 108 IU/ml) [8]. Concentrations of IgE antibodies to grass and birch pollen as well as total IgE levels were reported to be positively correlated with the soil levels of ^137^Cs [6-9]. Hence, it seems that long term exposure to ionizing radiation after the Chernobyl incident may result in higher IgE levels in children residing in contaminated areas. However, the prior studies did not take into account confounders such as cat and dog exposure that may be associated with the exposure to ^137^Cs in the soil and may affect serum IgE levels.

Findings describing serum levels of IgA, G, M, and E in children residing in ^137^Cs- contaminated areas are not directly comparable since they present changes occurring at different times after the nuclear incident, use either different laboratory tests for immunoglobulins [6,8] or do not report which tests were used [12,17,19], involve children of different age, use different exposure measures such as ^137^Cs soil levels or body burdens, and define exposures in different ways. We did not identify any studies characterizing immunoglobulins in children of different age who remained in the contaminated territories for a longer period of time. To enhance our knowledge about effects of radiation on the immune system, we investigated associations between long term exposure to ^137^Cs and levels of IgA, G, M and E in various cohorts and samples of children who resided in the Narodichesky district, Ukraine, for longer periods of time.

## Methods

### Study population

**Figure 1:**
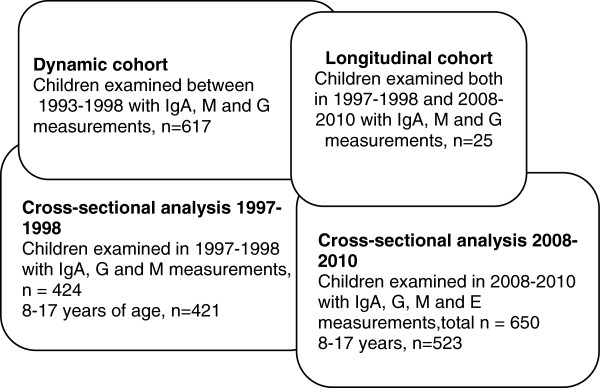
Cohorts and cross-sectional samples of the Narodichi Children Study used for the analyses.

Our investigation focused on three distinct study groups: 1) a dynamic cohort, 2) two cross-sectional samples, and 3) a longitudinal cohort (Figure 1). We consider this dynamic cohort investigation a natural experiment because the entire population was randomly exposed to radiation, although the exposure allocation was not controlled by the investigators. There are no differences in climate, temperature, wind, or other weather conditions between the villages in this area that may have affected the fall-out. Additionally, the fall-out was not related to differences in the resident population, occupation, or living conditions. Following the Chernobyl nuclear incident people residing in surrounding regions were exposed to different levels of ionizing radiation emitted by ^137^Cs and other radionuclides in a relatively random distribution independent of other social, physical, behavioral, and genetic risk factors. In addition, this is a dynamic cohort since not all children participated in all years; new children entered and older ones left the cohort due to reaching adulthood. The cohort is described in detail elsewhere [13,20]. The dynamic cohort comprises 616 children 3–16 years of age with repeated medical exams between 1993 and 1998.

To investigate changes in immunoglobulins between 1997–1998 and 2008–2010, we compared two cross-sectional analyses with different participants who were assessed at a similar age. The first cross-sectional subsample consists of 424 participants of the mentioned above dynamic cohort (421 were 8–17 years old) who had last physical examination in 1997–1998 and measurements of serum IgA, G and M (cross-sectional sample 1997–1998). The second cross-sectional subsample includes 650 children from the Narodichesky region examined in 2008–2010 (2008–2010 cross-sectional sample); of these, 523 children were 8–17 years of age. In the 2008–2010 cross-sectional sample we measured IgE for the first time and collected additional information on smoking exposure history, household exposures, asthma, eczema and rhinitis. Lastly, we identified a sample of 25 participants who were examined in 1997–1998 and reexamined 11 years later, in 2008–2010, and had measurements of IgA, G and M in both physical examinations (longitudinal study). In this cohort we can identify individual changes based on two measurements, 11 years apart.

### Assessment of exposure to ^137^Cs

The residential soil contamination levels of ^137^Cs were used as a proxy of a child’s exposure to ^137^Cs. The most important radioactive isotope is ^137^Cs, which is formed by nuclear fission, has a half-life of 30 years and emits beta and gamma radiation. Dosimetric assessments of settlements in Ukraine contaminated with radionuclides were published from 1991 to 2005 in 10 reports [21]. In addition, the Ministry of Emergency Situations provided spatial data on radiation contamination in the soil [22,23]. These values were used for exposure assessment for the dynamic cohort (1993–1998). For children examined in 2008–2010, data on soil, cow milk, and potato concentrations of ^137^Cs in the various locations were obtained from the report by the Ukrainian Ministry of Emergency Situations [22] and from the Ministry of Health of Ukraine [24]. For the cross-sectional subsamples (1997–1998 and 2008–2010) ^137^Cs soil contamination was grouped into quintiles previously established for the dynamic cohort of 1993–1998 [13]. Identical cut-off points, also established for the 1993–1998 investigation [13], were applied to categorize the soil contamination for the 25 participants of the longitudinal cohort.

In addition to residential soil contamination levels of ^137^Cs, the individual whole-body burden of ^137^Cs was measured in 2008–2010 with a gamma-spectrometer Whole Body Counter “SCRINNER-3M”, designed and produced by INECO (Ukrainian Institution of Human Ecology, Academy of Technological Sciences), and used for mass screenings in 12 medical centers of the Ukraine [25]. The technical characteristics of “SCRINNER-3M”, calibration and measurement protocol were described in detail elsewhere [26]. To preserve the longitudinal comparisons of the samples, we only used individual whole-body count of ^137^Cs for comparison of exposure measures. The body burden of ^137^Cs was adjusted for weight (kg) [27]. Because of known variations with season of quantification [28,29], we also took the month of measurement into account. After adjustment for weight, month, age, and gender of the child in linear models using log-transformed whole-body ^137^Cs, we estimated the residuals. For the residuals, which do not yet contain the effect of residential exposure, we tested their correlation with levels of ^137^Cs soil contamination (kBq/m^2^) and cow milk contamination (Bq/L).

### Measurements of immunoglobulins A, G, M, and E

In participants examined between 1993 and 1998 concentrations of immunoglobulins A, G, and M were determined in fasting serum samples collected in EDTA tubes using reverse radial immunodiffusion (Mancini technique). In participants examined in 2008–2010, total concentrations of IgA, G, and M in morning fasting serum samples were analyzed using enzyme immunoassay (RT 2100C Microplate Reader, Rayto, China) with ELISA immunokits Serazym IgG, IgM and IgA (Seramun Diagnostica GmbH, Berlin, Germany). To compare the Mancini technique and enzyme immunoassay, we measured total serum levels of IgA, G, and M in 20 children using both methods and calculated intraclass correlation coefficients (ICC) [30]. ICCs showed high comparability of the two methods: IgA: 0.99, IgG: 0.95, and IgM: 0.98. Concentrations of antigen-specific IgE in participants examined in 2008–2010 were analyzed using Alpha Rapid test (Dr. Fooke Laboratorien GmbH, Neuss and Neuruppin, Germany). We used two mixtures of the most common allergens: indoor (*Dermatophagoides pteronyssinus*, *Dermatophagoides farina*, *Aspergillus fumigates*, *Aspergillus niger*, cat and dog dander) and outdoor (seasonal weeds and flowers). Due to budgetary constraints, IgE could only be measured in 180 of the 523 children 8–17 years of age.

### Interview data

In the 2008–2010 investigation, we collected information about additional potential confounders, since behavioral adjustment to the accident may have been different in groups with different random allocation of radiation. Data included exposure to tobacco smoke at home, active smoking, and antibiotic use in the last 12 months, exposure to cats and dogs living in a household, exposure to coke, coal, wood and gas used for fueling the stove for heating and cooking. Information about asthma-, rhinitis- and eczema-like symptoms was obtained during the interview using the Russian version of the standardized International Study of Asthma and Allergies in Childhood (ISAAC) questionnaire [31]. For asthma, we elicited information about the following symptoms within 12 months prior to the interview: wheezing/whistling, wheezing attacks, sleep disturbances due to wheezing/whistling, and wheezing during/after physical activity. Regarding rhinitis in the last 12 months, data was collected on sneezing/running nose/nasal congestion not related to cold, nose problems accompanied by itchy-watery eyes, and information on whether nose problem interfered with daily activities. For eczema, we gathered answers about itchy rash in the last 12 months and whether it manifested in the folds of the elbows, behind the knees, in front of the ankles, under the buttocks, or around the neck, ears or eyes; whether the rash had cleared completely at any time during the last 12 months; and whether the child had been kept awake at night by this itchy rash in the last 12 months. For each manifestation, the sum of positive answers was scored: zero, one and 2–4 symptoms.

### Statistical analysis

We used four different epidemiological approaches (Figure 1): a dynamic cohort, two cross-sectional studies, and a small longitudinal study. For the dynamic cohort (1993–1998) and the small longitudinal study (from 1997–1998 to 2008–2010), linear mixed models [32] were employed to analyze the effect of residential soil levels of ^137^Cs on repeated measurements of IgA, G and M. To adjust the repeated measurements for within-participant effects, we used the regular maximum likelihood method and started with an unstructured covariance matrix, which requires the least amount of constraints. An autoregressive covariance matrix significantly improved the model fit based on the evaluation of Akaike information and Bayesian Schwarz information criteria [32]. The analysis of the dynamic cohort (1993–1998) concentrated on four main effects (soil contamination, child’s gender, child’s age, and year of the exam) and the interaction between year and soil contamination. We used quintiles of ^137^Cs soil contamination developed in 2008 [13].

The same categories were used for the longitudinal cohort of 25 participants, however, without the highest exposure levels. For this small sample we applied mixed linear models to analyze the two repeated measurements.

For the cross-sectional analyses of data for 1997–1998 and 2008–2010, we used linear regression models to assess the association between quintiles of soil contamination and IgA, IgG, IgM, and adjusted for child’s gender and age at the time of the physical examination. In addition, in the cross-sectional analysis 2008–2010, we adjusted for household and individual characteristics. All data analyses were performed using SAS software (Version 9.2: Statistical Analysis System, Cary, NC).

Immunoglobulin levels typically increase until the age of 11 years and then remain at a comparable level thereafter [33]. Hence, in the two cross-sectional studies, we stratified children into two age groups: 8–11 and 12–17 years, which allows us to compare corresponding groups in the two cross-sectional analyses. In the cross-sectional analysis of the 1997–1998 data we controlled for child’s age, gender, and year of investigation using general linear regression models. For the 2008–2010 data, we considered additional adjustment for exposure to environmental tobacco smoke (ETS), active smoking, antibiotic use, exposure to cats and dogs, and coal, coke, wood or gas used as a fuel in stoves for heating and cooking. Age was used as a categorical variable to take possible nonlinear effects into account. Children were considered exposed to environmental tobacco smoke (ETS) if they had direct or parental exposure to ETS at home, or if either parent was identified as a smoker. For children 12–17 years of age, we calculated the average number of cigarettes per day smoked by each child and categorized the variable into three groups: nonsmokers, 10 cigarettes per day or less, more than 10 cigarettes per day. Frequency of antibiotic use during the 12 months prior to the physical examination was grouped: no use, one time, two or more times. For exposures to cats and dogs living in the household we used three groups: none, cat/dog living outside and not allowed in the house, cat/dog living in the house or living outside but allowed to come inside. Exposure to coke, coal, and wood used as a fuel in the stove for heating or cooking was combined in one variable (yes/no). Exposure to gas fumes produced during cooking or heating was treated as a separate variable (yes/no). Regarding allergic manifestations, the sum of asthma-, rhinitis- and eczema-like symptoms was scored: zero, one and 2–4 symptoms. Confounding was assessed using 10% rule; i.e., a potential confounder was kept in the explanatory model if its removal changed the estimate between exposure and immunoglobulin by more than 10%.

## Results

From the comparison of the two cross-sectional studies it is obvious that children examined in 1997–1998 were comparable with children examined in 2008–2010 in regard to gender (51% versus 49% were female) and median age (12 versus 14 years, Table 1). The distribution of the ^137^Cs soil contamination changed considerably between 1997–1998 and 2008–2010. In 1997–1998, 39.6% resided in areas with the highest level of soil contamination (350 kBq/m^2^ and above). In 2008–2010, most children lived in areas with medium levels of soil contamination (165–265 kBq/m^2^). Only one child came from the area with the second highest soil contamination level (266–349 kBq/m^2^); this child was included in the medium exposure group. This led to three exposure levels for the years 2008–2010. The proportion of children in areas with the lowest soil contamination (<116 kBq/m^2^) increased only by 3.4% in 2008–2010. Overall, the median soil exposure in children did not decrease. The mean serum concentrations of IgA, IgG and IgM in children 8–17 years in the cross-sectional study 2008–2010 were 1.86 g/l, 11.6 g/l and 1.14 g/l, respectively.

**Table 1:** Characteristics of children with immunoglobulin values examined in various cohorts and cross-sectional samples

**Characteristics**	**Dynamic cohort, 1993-98 n = 617%**		**Cross-sectional analysis 1997-98 n = 424%**	**Longitudinal cohort 1997–98 and 2009-10**		**Cross-sectional analysis 2008-10 n = 650%**	
				**1997-98 n = 25%**	**2008-10 n = 25%**		
Girls (total)	51.5		50.9	52	52	48.6	
8 years & younger	40.6		0.5			9.5	
8-11 years	42.8		44.4			17.1	
12-17 years	16.7		55.1			61.1	
>17 years	0		0			12.3	
Boys (total)	48.5		49.1	48	48	51.4	
8 years & younger	44.1		1.0			7.8	
8-11 years	37.1		51.4			18.9	
12-17 years	18.7		47.6			63.8	
>17 years	0		0			9.6	
Age: median (5%, 95%)	8 (3, 14)		12 (8, 16)	11 (8, 14)	22 (19, 26)	14 (5, 22)	
Residential ^137^Cs exposure (kBq/m^2^)	Mean, (kBq/m^2^)	%				Mean, (kBq/m^2^)	%
<116	90.8	17.5	19.1	16.0	32.0	80.0	22.5
116 - 164	128.4	18.0	16.5	20.0	16.0	131.2	10.6
165 - 265	196.7	20.1	19.1	20.0	52.0	247.3	66.8
266 - 349	308.9	6.7	5.7	8.0	0	NA	0.2
≥350	354.9	37.8	39.6	36.0	0	NA	0
Residential ^137^Cs exposure, median (5%, 95%), kBq/m^2^	210 (79, 364)		224 (79, 364)	224 (112, 364)	171 (60, 253)	253 (60, 264)	

**Figure 2:**
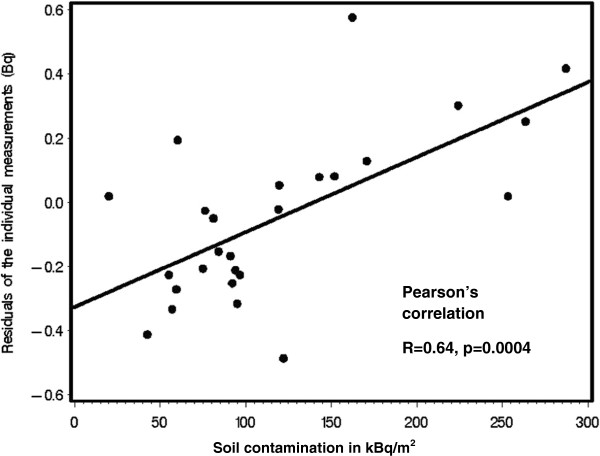
**Relationships between residuals (Bq) after prediction of individual**^**137**^**Cs body burdens and residential soil contamination.** Generalized linear models used to predict individual ^137^Cs body burdens included weight, age, gender and month of measurement.

Soil contamination values in 2008–2010 were only estimates based on measurements taken in 1992 and a decay function. Hence, to corroborate our findings for the exams in 2008–2010, we also used radiation levels in cow milk samples (Bq\L) collected in 2008 in different villages (n = 25; milk samples were collected yearly). Both soil contamination and cow milk samples from the different villages were strongly correlated (r = 0.7, p <0.0001, n = 25) which increases credibility of estimated soil exposure. In addition, using individual measurements of whole-body burden of ^137^Cs (continuous, Bq) in a linear regression model we adjusted for age (years), gender, weight (kg) and month of measurement, and determined the residuals. These were normally distributed. Then, the residuals of these individual measurements (not explained by residential exposure) were correlated with the area ^137^Cs soil contamination (kBq/m^2^). Indeed, the correlation in 26 villages was high (r = 0.64, Figure 2). Finally, using the same approach, we correlated the residuals of the individual whole-body burden of ^137^Cs with the radiation values in milk (Bq\L) for 2008. The correlation coefficient was lower (0.42, p = 0.04, 25 villages).

**Figure 3:**
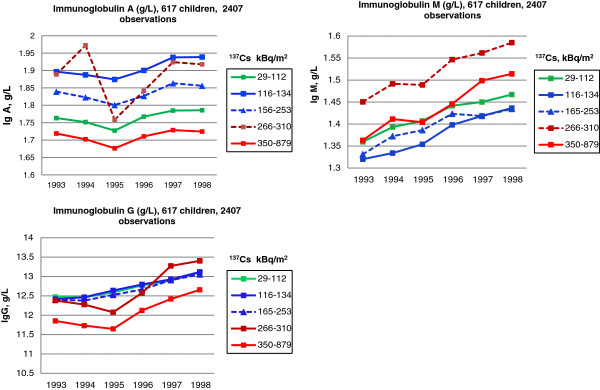
**Changes of immunoglobulins A, G, and M between 1993 and 1998 by **^**137**^**Cs exposure.** The residential ^137^Cs soil contamination is grouped into quintiles.

In the dynamic cohort, 617 children had repeated measurements between 1993–1998, providing 2,407 measurements of IgA, IgG, and IgM. In mixed linear models we predicted mean levels of each immunoglobulin (the outcome) based on quintiles of ^137^Cs soil contamination (the exposure) and adjusted for gender and age of the child, year of the exam, and the interaction between year and soil contamination. Regarding IgA, the highest and the lowest exposure groups (≥350 kBq/m^2^ and 29–112 kBq/m^2^, respectively) had the lowest immunoglobulin levels (Figure 3). Children in three groups with a median soil exposure had the highest IgA serum concentration. For IgA, the main effect of soil radiation and its interaction with the year of the exam were statistically significant (p = 0.034 and p = 0.039, respectively, Table 2). The lowest and two medium exposure groups (29–112 kBq/m^2^, 116–134 kBq/m^2^ and 165–253 kBq/m^2^, respectively) had very similar serum IgG concentrations which gradually increased over time. The lowest serum IgG level was observed in the group with the highest ^137^Cs soil contamination 350–879 kBq/m^2^. In the second highest exposure group (266–310 kBq/m^2^), serum IgG level dipped in 1995 and in 1997 exceeded levels for all other groups. For IgG, the interaction term between soil contamination and year were also statistically significant (p = 0.001). Regarding IgM, the highest serum IgM levels were in the third exposure group (266–310 kBq/m^2^; Figure 3). Although the interaction term between level of soil irradiation and year is statistically significant (p = 0.02), all exposure groups seem to show increased IgM serum concentrations over time (Figure 3).

**Table 2:** Statistics for linear mixed models applied to dynamic cohort 1993-1998

	**Numerator DF**	**Denominator DF**	**F-Value**	**Probability F-value**
**Immunoglobulin A (g/L)**				
Quintiles of the area irradiation: ^137^Cesium (kBq/m^2^)	4	1752	2.61	0.034
Gender	1	1752	0.77	0.38
Age groups	14	1752	2.66	0.0007
Year of measurement	5	1752	11.46	<0.0001
Quintiles of the area irradiation: ^137^Cesium (kBq/m^2^) × year	20	1752	1.63	0.039
**Immunoglobulin G (g/L)**				
Quintiles of the area irradiation: ^137^Cesium (kBq/m^2^)	4	1752	2.09	0.08
Gender	1	1752	3.26	0.07
Age groups	14	1752	3.62	<0.001
Year of measurement	5	1752	16.29	<0.001
Quintiles of the area irradiation: ^137^Cesium (kBq/m^2^) × year	20	1752	3.37	<0.001
**Immunoglobulin M (g/L)**				
Quintiles of the area irradiation: ^137^Cesium (kBq/m^2^)	4	1752	1.09	0.36
Gender	1	1752	0.04	0.84
Age groups	14	1752	2.47	0.002
Year of measurement	5	1752	5.09	0.0001
Quintiles of the area irradiation: ^137^Cesium (kBq/m^2^) × year	20	1752	1.73	0.023

Detailed legend: Linear mixed models were used to predict adjusted mean concentrations of immunoglobulins A, G and M in serum. Each model includes quintiles of residential ^137^Cs as a main exposure, child’s gender, age groups, year of the measurement, and the interaction term between quintiles of cesium soil contamination and the year of the measurement. Probability F-value shows statistical significance of each predictor in the full model.

*DF*: Degree of freedom.

While for the dynamic cohort (1993–1998) we assumed random allocation of radiation (natural experiment), for the children examined in 2008–2010 we needed to test whether potential risk factors differed in the three remaining exposure categories. Since immunoglobulins increase until age 11 years, we stratified the analysis by age (8–11, 12–17 years, Table 3). In both age strata, the frequency of antibiotic use within the last 12 months prior to physical examination did not differ among the exposure groups with various ^137^Cs soil contamination. In addition, we tested whether IgA, IgG, or IgM serum concentrations co-varied with antibiotic use in the last 12 months and found no association (data not shown). Antibiotic use was not confounding the association between residential exposures and immunoglobulins. Table 3 shows that children 8–11 years of age in the lowest ^137^Cs soil contamination category had significantly higher proportions of dogs and stoves fueled with coal, coke or wood in their households (Table 3). Similar findings were seen in the age-group 12–17 years. In addition, active smoking was significantly higher in older children residing in areas with the lowest ^137^Cs soil contamination (12.3% vs. 7.3%). Hence, it seems that there were potential confounders that could mask the ^137^Cs soil exposure and as a result multivariate adjustment was necessary.

**Table 3:** **Association between Cs**^**137 **^**residential soil contamination and confounders in children examined in 2008-2010**

**Characteristic**	**Exposure to **^**137**^**Cs in kBq/m**^**2**^			**P-value**	**Exposure to **^**137**^**Cs in kBq/m**^**2**^			**P-value**
	**<116, %**	**116-164, %**	**165-265, %**		**<116, %**	**116-164, %**	**165–349**^**a**^**, %**	
	**Children 8–11 years of age**				**Children 12–17 years of age**			
Antibiotic use during past 12 months								
Two or more times	5.6	13.3	2.4	0.082^b^	3.1	2.7	3.3	0.7426^b^
One time	33.3	20.0	15.5		9.3	16.2	14.2	
None	61.1	66.7	82.1		87.6	81.1	82.7	
Stove fueled with coal, coke or wood	50.0	0	23.8	0.002^b^	42.3	40.5	23.2	0.0005^c^
Gas from the mains used for cooking	88.9	100.0	95.2	0.378^b^	88.7	97.3	91.2	0.3119^b^
Cat exposure								
2^d^	44.4	20.0	31.0	0.205^b^	29.9	37.8	44.1	<.0001^c^
1^e^	50.0	80.0	53.6		57.7	54.1	31.6	
0^f^	5.6	0	15.5		12.4	8.1	24.3	
Dog exposure								
2^d^	5.6	0	1.2	0.002^b^	5.2	0	4.0	0.0032^b^
1^e^	94.4	86.7	61.9		85.6	81.1	69.5	
0^f^	0	13.3	36.9		9.3	18.9	26.5	
ETS	55.6	40.0	38.1	0.392^c^	49.5	40.5	46.0	0.6369^c^
Number of cigarettes smoked per day by a child								
0					87.7	92.8	95.7	0.0001^b^
10 or less					11.6	2.9	2.3	
More than 10					0.7	4.4	1.8	

^a^Quintiles 165–265 and 266–349 kBq/m^2^ were combined since there was only one child in group 266–349 kBq/m^2^.

^b^Fisher’s exact test.

^c^Chi-square test.

^d^Cat or dog lives in the house or lives outside but comes inside.

^e^Cat or dog lives outside and not allowed inside.

^f^No cat or dog in the household.

Using general linear models, we did not find statistically significant associations between categorized ^137^Cs soil contamination and immunoglobulin levels in the cross-sectional analyses of the 1997–1998 data (data not shown). However, in children 8–11 years of age examined in 2008–2010 after adjustment for age, gender, and environmental smoke exposure, the mean level of IgA was significantly higher in the ^137^Cs soil contamination category of 165–265 kBq/m^2^, representing the median exposure group of the 1993–1998 investigations (Table 4). In these children, levels of IgA were also significantly positively correlated with the levels of ^137^Cs in locally produced cow milk adjusted for the same confounders. In children aged 12–17 years, there was no significant association between soil contamination levels of ^137^Cs and IgA (data not shown). Immunoglobulins M and G were not associated with soil contamination levels of ^137^Cs in age group 8–11, nor in group 12–17 (data not shown).

**Table 4:** Concentrations of serum immunoglobulin A in children 8–11 years examined in cross-sectional study 2008-2010

**Model 1**	**Adjusted mean**^**a**^**in g/L (95% CI)**	**p-value**^**b**^	**Model 2**	**Adjusted mean**^**a**^**in g/L (95% CI)**	**p-value**^**b**^
^137^Cs in soil, kBq/m^2^			^137^Cs in milk, Bq/L		
<116	1.76 (1.43-2.09)	0.0067	≤15	1.68 (1.41-1.96)	0.0265
116 – 164	1.25 (0.88-1.63)^*^		16-85	1.60 (1.34-1.86)	
165 - 265	1.95 (1.77-2.13)		>85	2.02 (1.81-2.23)^*^	
Gender			Gender		
Female	1.64 (1.40-1.88)	0.8741	female	1.77 (1.56-1.97)	0.9934
Male	1.66 (1.46-1.86)		Male	1.77 (1.68-1.96)	
ETS			ETS		
Unexposed	1.71 (1.49-1.92)	0.4283	Unexposed	1.81 (1.63-1.99)	0.5436
Exposed	1.60 (1.37-1.83)		Exposed	1.73 (1.51-1.94)	
Age			Age		
8	1.30 (0.96-1.65)	0.0295	8	1.51 (1.19-1.82)	0.1189
9	1.99 (1.70-2.27)		9	2.04 (1.75-2.33)	
10	1.67 (1.35-1.98)		10	1.77 (1.45-2.08)	
11	1.65 (1.38-1.91)		11	1.76 (1.54-1.98)	

*ETS*: Exposure to environmental tobacco smoke; *CI*: Confidence interval.

^a^Linear regression model adjusted for gender, age and ETS. Exposures to cats, dogs or indoor heating mode were not confounding and did not need to be controlled for.

^b^F-test in general linear models.

^*^p < 0.05.

In the 2008–2010 cross-sectional study, specific IgE for mixtures of specific indoor and outdoor allergens were measured in a subsample of 180 children. Cross-tabulation of IgE reactions with ^137^Cs soil contamination did not detect any positive response in the exposure category of 116–164 kBq/m^2^, which was therefore combined with the 165–265 kBq/m^2^ category. Controlling for age, gender, dog and cat exposure, in logistic regression analyses, IgE responses to indoor allergens were statistically significantly lower in children residing in the 116–265 kBq/m^2^ area (p = 0.013, Table 5). There was no statistically significant difference for IgE specific to outdoor allergens.

**Table 5:** **Residential **^**137**^**Cs soil density and IgE responses to allergens in cross-sectional sample 2008-2010**

**Residential **^**137**^**Cs soil levels (kBq/m**^**2**^**)**	**Positive specific IgE reaction (row percentage)**	**Odds Ratio**^**a**^	**5%-95% CI**
	**Indoor allergens**		
< 116 (n = 37)	16.22	1.00	Ref.
116-265 (n = 143)	3.50	0.20	0.05-0.71
	**Outdoor allergens**		
< 116 (n = 37)	10.80	1.00	Ref.
116-265 (n = 143)	8.40	0.70	0.21-2.40

*CI*: Confidence interval, *Ref.:* Reference category.

^a^Logistic regression model adjusted for child’s gender, age, dog and cat exposure.

To explore whether other factors influence immunoglobulins, we investigated three symptom groups of allergic sensitization manifesting as asthma-like, rhinitis-like and eczema-like symptoms in children ages 8–17 examined in 2008–2010 (n = 523). We did not detect any association between ^137^Cs soil contamination and the symptom groups since asthma-like (1.7%), eczema-like (3.6%) and rhinitis-like symptoms (3.5%) were reported only for a few children. Having symptoms from any of the three groups was not related to changes in immunoglobulins A, G, and M.

The follow-up study of 25 participants (longitudinal cohort) examined in 1997–1998 and reexamined in 2008–2010 allowed us to investigate changes in immunoglobulins A, G, and M in a small longitudinal sample. The age range at the first medical exam was 8–15 and 19–27 years in the second. The median soil contamination level of ^137^Cs decreased from 224 to 171 kBq/m^2^ (Table 1). Due to the small sample size, we considered only three exposure levels (29–112 kBq/m^2^, 116–165, and > 265 kBq/m^2^). In 1997–1998, 4, 5, and 16 children were in the three respective groups; in 2008–2010 the numbers were 8, 4, and 13.

In this longitudinal cohort, serum IgA levels did not change. The individual plots of IgG for 25 longitudinal observations over 11 years showed substantial dynamics: the lower levels seemed to increase and the higher to decrease (Additional file 1). The result of mixed linear regression revealed that in the higher exposure groups, IgG increased from time 1 (1998–1997) to time 2 (2008–2010) from approximately 9.5 g/ml to approximately 14 g/L (Table 6). It remained more stable in the low exposure group (approximately 13.1 g/L and 11.9 g/L). On the contrary, IgM decreased in the group of children to adults with the highest residential exposure (165–364 kBq/m^2^) from 1.36 g/L to 0.94 g/L and remained more stable in the other two groups.

**Table 6:** Serum immunoglobulins G and M in a small cohort examined in 1997–1998 and 2008-2010

**Residential **^**137**^**Cs soil levels (kBq/m**^**2**^**)**	**Time period of the medical exam**		**p-value (F-test) for the interaction of exposure and exam year**
	**1997-98**	**2008-10**	
	**Adjusted mean in g/L (5%-95% CI)**^**a**^	**Adjusted mean in g/L (5%-95% CI)**^**a**^	
**IgG**			
165 – 364	9.20 (7.42-10.98)	14.79 (13.15-16.45)	0.009 (6.86, Numerator DF = 2, Denominator DF = 13)
116 – 164	9.72 (7.06-12.39)	13.80 (11.31-16.29)	
< 116	13.10 (10.81-15.39)	11.91 (9.92-13.89)	
**IgM**			
165 – 364	1.36 (1.20-1.52)	0.94 (0.79-1.09)	0.001 (63.05, Numerator DF = 2, Denominator DF = 13)
116 – 164	1.27 (0.98-1.55)	1.31 (1.15-1.46)	
< 116	1.38 (1.22-1.54)	1.34 (1.19-1.50)	

Detailed legend: Linear mixed models used to predict adjusted mean serum levels of immunoglobulins G and M in 25 persons who were examined 2 times, in 1997–1998 and in 2008–2010. The main exposure is tertiles of ^137^Cs in the area of residence.

*DF*: Degree of freedom; *CI*: Confidence interval.

^a^Adjusted for gender, age, and year of the medical exam.

Interestingly, between 1993 and 1998, IgM levels particularly in the two highest exposure groups (266–310 and 350–879 kBq/m^2^) increased (Figure 3, sample size n = 617). However, in the small longitudinal cohort (n = 25) with measurements in 1997–1998 and 2008–2010, IgM levels decreased in the highest exposure group (165–253 kBq/m^2^). The latter group had the least increase from 1993–1989 (Figure 3). However, both trends may not be comparable since the age in the 1993–1998 cohort was 1–17 years and in the longitudinal cohort it was 8–15 years at the first medical exam and 19–27 years in the second.

## Discussion

Our data suggest that immunoglobulins were still affected 6–12 years after the nuclear incident and remained affected more than 20 years later. There is no clear linear pattern, but various convex-curve-like associations. IgA appears to be higher in children with the medium exposure level (dynamic cohort 1993–1998) compared to children with lower and higher exposures (convex curve). IgG seems to gradually increase in children with lower exposure levels (dynamic cohort and longitudinal cohort), however, in two groups with the highest exposure, IgG first declines and then increases approximately 9 years after the incident. IgM was also found to be higher in the medium exposure groups (dynamic cohort) and lower in children with the higher residential exposure (longitudinal cohort). IgE specific to indoor allergens was lower in children with higher residential exposure.

Using various approaches, we attempted to establish and analyze follow-up data of the Narodichi Children Cohort (NCC) from 1993 to 2010. We used ^137^Cs soil contamination levels, which were available for all investigations between 1993 and 2010. The availability facilitates comparisons over time; however, the disadvantage of using spatial exposure assessments is that it may result in less precision compared to measurements in individuals. Adding to this uncertainty, we realized that the levels documented after 1992 were estimated based on measurements in 1992 and the decay function (^137^Cs: half-life of 30 years) and not actual measurements. Nevertheless, among 26 villages where children had whole body counts, the individual and the soil radiation levels (2008) correlated reasonably well (r = 0.64). A comparable correlation (r = 0.7) was reported in the past [34]. Radiation in cow milk from different villages was measured repeatedly since 1992. The residuals of individual measurements, after adjusting for age, body weight, and month of measurement, and the radiation concentration in cow milk were also correlated (r = 0.42). Given the residential stability of the children and the lack of individual measurements before 2008, soil measurements in various villages seem to constitute acceptable radiation exposure assessments and have the advantage of long-term comparability, which is needed to evaluate changes in outcome variables.

In the dynamic cohort 1993–1998, for all immunoglobulins, A, G and M, a similar secular trend was observed with dip or plateau around 1995 and consequent increase over time (Figure 3). Reduced levels in 1995 may be related to changes in supply of radiation-free food provided by the Ukrainian government to residents of radionuclide contaminated areas after the Chernobyl incident. In 1995, due to deteriorated financial situation, the Ukrainian government stopped delivery of uncontaminated food to the residents, and radionuclide-free meals at schools were reduced down to two per day. As a consequence, the residents increased their consumption of locally grown produce and forest goods contaminated with ^137^Cs. Technical causes that might contribute to these changes are unlikely, since during this period, laboratory analyses were conducted by the same personnel using the same techniques.

For the dynamic cohort 1993–1998, we discovered that the serum IgA concentrations did not change much over time; children residing in areas with medium level of ^137^Cs soil contamination (116–310 kBq/m^2^) had higher IgA serum concentrations compared to children in villages with lower (<116 kBq/m^2^) and higher (>350 kBq/m^2^) exposure levels (convex-curve-like association). The main function of IgA is neutralization of antigens on mucous membranes including gastro-intestinal tract [35]. We hypothesize that higher serum levels of IgA in medium exposure groups as compared to low exposure may reflect an increased response of the immune system to ^137^Cs ingested with locally produced food, which is now considered the main way how ^137^Cs enters human body [36,37]. On the contrary, exposure to higher levels of radiation may have slightly decreased the immune response; therefore, the levels of serum IgA in these children were lower than in all other exposure groups and in agreement with the previous studies [5,6]. The findings in the dynamic cohort 1993–1998 were corroborated in the 2008–2010 data in children 8–11 years. Children residing in areas with a soil contamination level of 165–265 kBq/m^2^ (medium exposure group in 1993–1998 cohort and the highest in 2008–2010) and consuming cow milk with higher ^137^Cs levels (>85 Bq/L) had higher levels of serum IgA as compared to the lowest exposure levels even 25 years after the incident. This is in agreement with investigations conducted in Belarus [17] and in residents of villages located on the riverbanks of the Techa River [38]. The Techa River was contaminated as a result of radioactive releases by a plutonium production facility in 1949–1956. Residents with continuous exposure to ionizing radiation showed significant increase in serum levels of IgA at all levels of dose without a definite trend. Our findings further agreed with the analysis of yearly measured radiation levels in cow milk (Bq/L) from the different villages, indicating a convex-curve-like association.

Regarding IgG and IgM, we found a significant increase in all residential exposure groups between 1993 and 1998 which is in agreement with the previous prospective study that evaluated serum immunoglobulin levels in children residing in contaminated areas of Belarus between 1986 and 1992 [6]. The longitudinal comparison of a small group of 25 participants between 1997–1998 and 2008–2010 showed that in participants with higher exposures, IgG levels increased, and IgM levels decreased in the highest exposure group (165–364 KBq/m^2^). The higher concentrations of IgG in serum in response to continuous exposure to low-dose ionizing radiation might be explained by inherent features of IgG. Due to its small size, IgG easily diffuses into tissues, opsonizes pathogens for engulfment by phagocytes, activates the complement system, and neutralizes antigens [39]. We speculate that IgG may be involved in neutralization of ^137^Cs which passed through the intestinal mucosa into the systemic circulation and reached tissues. Regarding IgM, the largest among the immunoglobulins and the first one to interact with new antigens [39], we speculate that in children exposed to ^137^Cs for a long period of time, the serum IgM levels could have been affected by decreased immune response.

We believe that selection bias did not distort our findings. After the Chernobyl nuclear incident all children residing in the Narodichesky district of Zhitomir Oblast, Ukraine, were obligated by law to participate in yearly health screenings, although it was never enforced. The incentive to participate in the screening was free hospital treatment at the Research Center for Radiation Medicine (Kyiv, Ukraine) provided to children with health problems. The trust and the excellent rapport between the team of physicians from the Research Center for Radiation Medicine and the pediatric clinic in the Narodichi hospital facilitated eager participation. All children from remote villages were transported by bus to the pediatric clinic in Narodichi. It is possible that a small number of sick children were not able to attend the screening.

Regarding immunoglobulins, the agreement between two measurements of immunoglobulins used between 1993 and 1998 and in 2008–2010 is high. Hence, there is no indication of a differential information bias.

Since in a prior report we found obstructive and restrictive lung function changes in the NCC [20], we added measurements of specific IgE against indoor and outdoor allergens to the set of clinical examinations conducted in 2008–2010. To our surprise we found that IgE specific against indoor allergens, but not outdoor allergens, was statistically significantly less frequent in children residing in villages with higher soil concentrations. In addition, the frequency of asthma-like (1.7%), eczema-like (3.6%), and rhinitis-like (3.5%) symptoms in children of the NCC is low compared to northern and eastern European Countries [31]. Similar differences in the prevalence of these symptoms have been reported between the Finnish and Russian Karelia [40]. Except for ‘western lifestyle’ no substantial explanation has been offered to explain this divergence. Of interest, is that these results suggest that allergic sensitization (positive specific IgE) against indoor allergen mix, but not outdoor allergens (pollen), is negatively associated with ^137^Cs soil contamination levels (OR = 0.02; 95%CI 0.05-0.71). It may be possible, that ^137^Cs radiation reduces the population of mite and mold species, reducing the indoor allergen but not outdoor allergen burden and thus the respective sensitization. This explanation is supported by findings that microorganisms in the feathers of birds in the Chernobyl region may be reduced by radiation [41].

## Conclusion

Effects of low-level radiation on the humoral immune system are detectable 20 years after the nuclear incident in Chernobyl (1986). Thousands of peasant children live in areas where the soil is still profoundly contaminated with ^137^Cs and consume locally grown foods. Surprisingly, higher soil radiation levels were related to fewer indoor allergen sensitizations (specific IgE), a finding, that needs to be replicated in future studies. The changes in the immunoglobulins cannot be seen as indication of increased risks leading to the manifestation of known adverse health effects, but should be considered as compensatory mechanisms in children coping with low-level radiation. There is a need to understand whether these compensatory mechanisms may fail, which could lead to adverse health effects. In addition, following the analyses of humoral immune responses, cellular immune responses and their interplay need to be analyzed. In summary, the results of this study point to the need for further surveillance, scientific evaluation, and continued environmental remediation.

## Abbreviations

137Cs: ^137^Caesium; CI: Confidence interval; DF: Degree of freedom; EDTA: Ethylenediaminetetraacetic acid; ELISA: Enzyme-linked immunosorbent assay; ETS: Environmental tobacco smoke; ICC: Intraclass correlation coefficient; Ig: Immunoglobulin; INECO: Ukrainian Institution of Human Ecology, Academy of Technological Sciences; ISAAC: International Study of Asthma and Allergies in Childhood; NCC: Narodichi Children Cohort.

## Competing interests

The authors declare that they have no competing interests.

## Authors’ contributions

DM reviewed the literature, analyzed the data, drafted and revised the manuscript. WK obtained funding, developed the analytical plan, analyzed the data, and revised the manuscript. YS and VV designed the study protocol and conducted clinical examinations and blood analyses. VK designed questionnaire and conducted the interviews. ES analyzed the data and revised the manuscript. OL collected dosimetric information. All the authors approved the final version of the manuscript.

## Supplementary Material

Additional file 1 Individual changes of IgG levels between 1997–1998 and 2008–2010 in the longitudinal cohort of 25 participants. Click here for file

## Study approval

The investigations were approved by the Committee on Bioethics of the Research Center of Radiation Medicine Academy Medicine of Sciences of Ukraine (Report #10, August 31, 2007) and by the Office of Research Compliance at the University of South Carolina.
