# Supplementary figures and images for: Effects of long-term low-level radiation exposure after the Chernobyl catastrophe on immunoglobulins in children residing in contaminated areas: prospective and cross-sectional studies
Source: Environ Health. 2014 May 10;13:36. doi: 10.1186/1476-069X-13-36 (PMC4030024; doi:10.1186/1476-069X-13-36)

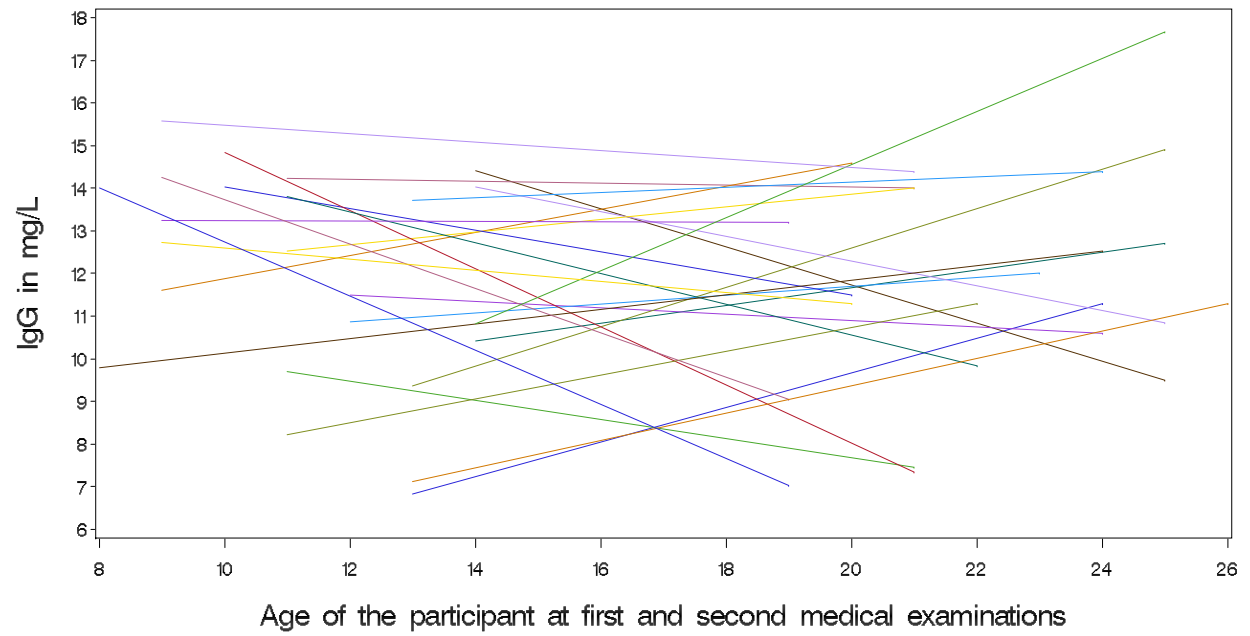

Supplement: Additional file 1 — Individual changes of IgG levels between 1997–1998 and 2008–2010 in the longitudinal cohort of 25 participants. [file 1476-069X-13-36-S1.pdf]
